# Nervous Influence
†Enquiry into the Nature and Effects of the Nervous Influence, and its connexion with the Vital, Moral, and Intellectual Operations. By Mrs. Carleton. Galignani, Paris. Baillière, London. 8vo. pp. 238.


**Published:** 1852-04-01

**Authors:** 


### ART. III.—NERVOUS INFLUENCE.†

THIS is a remarkable book. An English work on Psychology, printed in Paris in 1836, and written by an English lady, is not likely to attract attention in 1852, unless it has considerable merit; and we confess we took it up from the table of a friend who knew and valued the authoress with that amount of scepticism as to its merits with which those who are trained to any calling are apt to view the productions of amateurs, and in addition with that doubt as to the probability of a lady treating physiological questions with any other foundation than the uncertain one of a knowledge of words and not of things. A hasty glance at a few pages induced us to borrow it, and a *second* perusal of the whole volume (the best compliment a reader can pay a writer) was the preparation for this review. That a book with such a title, without the name of the writer,

† Enquiry into the Nature and Effects of the Nervous Influence, and its connexion with the Vital, Moral, and Intellectual Operations. By Mrs. Carleton. Galignani, Paris. Baillière, London. 8vo. pp. 238.

printed in Paris, and not advertised in this country, should have remained unknown for a long period, is to be expected; but we have no such superabundant supply of sound, well-considered, and original thought, in the present day, to justify our still neglecting it; and we are simply doing justice to its gifted authoress, and a real service to those of our medical brethren who take some delight in psychology, by informing them of its existence. After reading it, few will (we believe) deny that among the able professional writers of the day, there are many who can put their contemplations on man's mind and its connexion with bodily states, in clearer, simpler, fuller, choicer language; who can clothe their thoughts in a purer or more graceful style: most professional writers have a larger amount of acquired knowledge, and a more extended acquaintance with the whole subject, owing to the care with which physiological facts have recently been both accumulated and diffused; but few bring to their materials the power of a more deeply contemplative mind, or show higher ability to think for themselves. There are certain defects of scientific form which many could gloss with more circumlocution; but those who judge a book by its merits and not by its defects, and who know the difficulty of the subject, will, we are sure, pass but one judgment upon it. From the book having been printed in Paris the errata are numerous, and a mistake has been made in placing as heads of divisions what were intended to be marginal notes of the subjects, but essentially there is much order and clearness of arrangement. The first part contains a theory of nervous and vital actions, which, considering it was the work of a woman, and was written many years previously to the date of publication, and that was sixteen years ago, shows the writer to have approached very nearly to those views which sound thinkers are now adopting, and to be gifted with insight into deep physiological principles; an insight which, had it been trained by experiment, would have probably produced an original physiological discoverer. The writer candidly brings these views forward merely as opinions which may eventually be proved to be correct by experiment. Her theory is a bold, a large, and an ingenious one, and we take it to be this: That the nervous power is analogous to the electric power, and that this nervous power as well as the other vital powers are kept in action by a constantly acting external stimulus, which is electricity; that it is the electricity, combined with the oxygen gas we breathe, as well as the uncombined electricity in the air, which is the effective vital stimulus; and that animal heat is an electrical operation also, because it is a chemical one; for all chemical changes (according to Sir H. Davy) are owing to the union of opposite electricities. This was written, we believe, nearly thirty years ago, and we will compare it with the view to which those physiologists have arrived who are conversant with modern physical discovery, as well as with physiological science. Faraday has proved the truth of Davy's hypothesis, that galvanic action is merely chemical action, acting in a continuous line returning into itself, instead of between particles at insensible distances. He has proved experimentally that galvanism and electricity are one and the same thing. Now, if chemical action will produce galvanism, galvanism, as is well known, will produce heat, light, magnetism, motion, and chemical decomposition; and, what is more curious, either of these in its turn will produce all the rest. Professor Grove, who has most ably theorized as well as experimented on this subject, regards all these forces as correlated, or having the relation of mutual dependence.

If, then, chemical action is thus convertible into galvanism, magnetism, heat, light, and motion, there is no difficulty in conceiving that in the body it is convertible into vital actions, and that the various vital forces are mutually dependent on the physical forces, as these are dependent on each other. Dr. Carpenter has ably followed out Professor Grove's theory, and has shown that the vital forces are convertible into the physical forces, as these are into the vital. By this theory it is not affirmed that the vital forces are the same as the physical, but that they are correlatives, or virtually dependent. As electricity is convertible into magnetism, but is not magnetism, so chemical action may be converted into nervous action, but is not nervous action. It is clear, therefore, that there is no discrepancy, but a remarkable agreement, between the theory of our authoress and the present doctrine. In both, the vital actions, and especially heat, are regarded as the sequence of chemical actions, and these chemical actions are electrical phenomena.

There are two points in this theory well worthy a strict experimental investigation, and these are, the influence of the uncombined electricity in the air we breathe on the body, and also the effects in health and disease of that magnetic atmosphere in which we live. The following observations on this latter subject are suggestive:—

"I am inclined to think that the various nervous states of the body at different periods of the twenty-four hours, are connected with the variations in the magnetic force of the earth at such times. It has been ascertained by Professor Hanstein, that the magnetic intensity of the earth is subject to a diurnal variation, decreasing from day-break till ten or eleven o'clock A.M., when it reaches its *minimum*, and from thence it increases until it reaches its *maximum*, about three o'clock A.M.

"Now I have observed that morbid affections which arise from *too great* an irritability in the system, as catarrh, fever, &c., increase in violence towards the time that this magnetism is rising to its maximum, and this period being passed, viz., three o'clock A.M., sleep and perspiration will succeed to the heat and restlessness of the first part of the night. I have also observed that in some complaints arising from languor and a *deficiency* of nervous action, the distressing feelings produced by it have been most apparent when the magnetism was at its minimum, and that the strength and spirits have risen when it was advancing to its maximum, after which the inclination to drowsiness has returned. These facts I have noticed in some very marked cases for months together. The increased rapidity of the circulation and development of heat towards evening, cannot, I should think, be attributable to the state of the digestive organs after a full or late meal, for it takes place independently of this circumstance, both in the healthy and feverish state, and in the latter, the little nourishment which is taken is frequently no greater in quantity at one period of the day than the other."

The second part treats of the connexion of the nervous influence with the mental operations. At a time when materialism was the fashion, our authoress, whilst recognising that the material part of our nature is indispensable in this state of existence to the performance of the mental functions, clearly distinguishes the two principles:

"One, dignified in its nature, unknown in its essence, characterized by the three general powers of *feeling*, *willing*, and *understanding*: the other subservient to the former, constituting the materials upon which it acts, and the tools by which it operates, and possessing at the same time the capability of acting upon and influencing it to a certain degree."

The emotions, feelings, passions, all that is commonly termed the heart, being that part of woman's nature which is most highly developed, is, as might be expected, ably treated. The feelings include the most material as well as the most spiritual part. They are, as a whole, most intimately connected with the body; more evidently so to the consciousness than the intellectual faculties. Mrs. Carleton distinguishes clearly the physical and moral sensations, the physical being the action of the nerves of sensation from without inwards, excited by external stimuli; the moral sensations being similar nervous actions called out by ideas of the mind. But why—and what physiologist has not often asked himself this question—why are those feelings, emotions, passions, which are included in the term moral sensations, located commonly in the heart? Even our harder, less impressible sex occasionally feel, or have felt, that emotions call out sensations in the left side of the chest, that we can localize them there with even more certainty than we can fix the seat of thought in our foreheads. From watching in herself these feelings, Mrs. Carleton, with much ingenuity, and, we think, truth, refers the sensation to the par vagum. The conscious feeling, the distribution of that nerve coming directly from the brain, and the effect of these emotions on the voice, heart, and stomach, render it very probable that it is the channel by which those thoughts which call out emotions and passions affect the vital organs of animal life, and explain why these feelings are popularly referred, not to the head, in which they undoubtedly originate, but to the heart. Mrs. Carleton describes a form of nervous disease which simulates successively an affection of the lungs, of the heart, and of the stomach, and which is attended with a potent effect on the feelings, irascibility, agitation, melancholy, anxiety, arousing passions which do not belong to the character, and which she refers to an excited state of this nerve in those of ardent temperament; whilst in the phlegmatic and hypochondriacs, the habitual melancholy is considered to be owing to a deficient excitability of the same nerve. We direct attention to a lengthened description of this affection, as it is evidently drawn from close (and probably self) observation, and on this account valuable, though the seat may not be admitted.

It is the third part, which is devoted to the effect of nervous influence upon the moral and intellectual character, where Mrs. Carleton is most at home, and which would have probably commanded much attention had it been addressed to the general public. Throughout, the close union of the nervous system and the mind is insisted on without any approach to materialism, and this union and the varieties in power of the two principles in different individuals are made the foundation for the distinction of temperaments.

Mrs. Carleton recognises an original mental as well as physical constitution; but, as in every mental operation two powers must combine, one physical and the other intellectual, the difference in the strength of these powers in themselves and relatively, is one source of variety of character, and in this difference she founds the distinction of temperaments. Her views on this subject are evidently founded on original observation and are true to nature. We do not say that they include all varieties of temperament, but they describe accurately four varieties of the nervous temperament.

"The strength or feebleness of the nervous action produces two temperaments, which I shall distinguish by the appellation of the ardent and the phlegmatic: each of the temperaments may be united to a strong or a feeble *intellectual power*, and these four combinations, with the several gradations from one extreme to the other, form the varieties of the natural mental constitution."

"ARDENT TEMPERAMENT.

"An energetic nervous action (which I am inclined to attribute to an abundant secretion of the nervous fluid,) produces a rapid circulation of the blood, a quick evolution of animal heat, with some tendency to inflammatory diseases, a certain degree of muscular power (independently of the strength or weakness of the muscular *fibre*), and a sensibility of the nerves, which gives vehemence to the feelings, warmth to the temper, and quickness and acuteness to the senses."

"PHLEGMATIC TEMPERAMENT.

"The slow nervous action is shown by a tranquil circulation, a low temperature of the blood, a moderate portion of physical strength, an absence of irritability in the nerves, and consequently in the temper."*

"COMBINATIONS OF THE PHYSICAL CONSTITUTION.

"The ardent temperament may be united, first, to a firm muscular fibre; and secondly, to a lax muscular fibre; and the phlegmatic temperament may also be combined with a strong or weak muscular system. Of all the constitutions, the ardent temperament combined with the lax muscular fibre is the most irritable: its physical strength is entirely derived from the vigour of the nervous action, and this is often irregularly distributed, and subjects the frame to various morbid affections, particularly of the nervous kind: the sensations are acute, and the mind partakes of the sensibility of the body, and is very liable to a morbid degree of irritability. The ardent temperament combined with a firm fibre, exhibits the greatest degree of physical strength: the constitution is vigorous, but liable to inflammatory diseases. The phlegmatic constitution united to a lax fibre exhibits the greatest deficiency of physical strength, but it does not seem particularly liable to disease until the strength is reduced below its natural standard by external circumstances: the nerves are not irritable, consequently the sensations are not acute, and the mind is usually placid. The phlegmatic temperament united to the firm fibre is the most desirable of all constitutions, as it gives the advantage of strength, without irritability: an athletic form, robust health, and an even temper are its usual concomitants."

"CLASSIFICATION.

"In enumerating the various combinations of the mental and physical qualifications with the two temperaments, I shall class them under the four following heads: 1st, the strong intellect combined with the ardent temperament; 2nd, the same united to the phlegmatic temperament; 3rd, the weak intellect combined with the ardent temperament; 4th, the same united to the phlegmatic."

Ardent temperament with weak intellect may deceive, from the quickness and facility of the nervous operations, but "the test of a good understanding is the reasoning faculty;" and the distinguishing characteristic of this temperament is "a *natural* want of judgment."

* "The richer the blood is in red globules, the stronger is the vital power, and the power of producing heat in the system. In the temperaments which physicians call *lymphatic*, in opposition to *sanguine*, and which I call phlegmatic, on account of its influence on the temper, the blood contains fewer of the globules which give it colour. It is more cold and watery, hence probably results the fairness of hair and skin, which is the usual external token of this temperament."

"Of all mental constitutions that which unites *weakness* in the immaterial principle, and *strength* in the nervous action, is the least calculated for its own happiness, or that of others; for it is subject to the greatest excess and variety of painful sensations, both mentally and bodily, with the fewest means of defence, that is, with the smallest share of firmness to control the one, and of patience to allay the other. The mutability of the human feelings, also, is particularly manifested in this character. Steadiness depends more upon the regulating power of the immaterial principle, than upon the nature of the feelings themselves—if the impulse of the present moment is habitually obeyed without reference to a settled line of conduct, no dependence can be placed upon the principles or affections, changeable in their direction as the waves of the watery element; without solidity, without a fixed foundation, the affections of a weak mind are at the mercy of every gale that blows: if the tide turns, it flows perhaps as strongly in an opposite direction, and the bitterest hatred succeeds the tenderest love. In short, instability is the characteristic of *mere* feeling. Maternal love alone forms an exception: *this* lies imperturbable in the hidden depths of the human heart, beyond the reach of the warring elements that disturb the surface.

"The errors which result from the weakness of the mind may be traced—1st, to an incapability of taking a general and extended view of things; 2nd, to a liability to be deceived by external appearances; 3rd, to a limited power of acquiring knowledge and of applying judiciously what *is* acquired. Those which are the consequences of immoderate activity in the sensitive department are to be traced, 1st, to hastiness of decision; 2nd, to the formation of strong prejudices; 3rd, to the habit of judging of the feelings of others by our own. Knowledge and experience are indispensable to an individual of this temper, so liable to err, so often blind to his own failings, and so exquisitely susceptible of suffering from their evil consequences."

In the ardent temperament with strong intellect, is found the highest intellectual perfection, "for both the material and immaterial parts contribute to the production of the talents." As, however, the feelings are also powerful, moral perfection, though not incompatible, is not a natural attendant. Great vices exist here as well as great virtues. To such a mind, sound religious principle is indispensable. We have not space to quote the admirable sketch of the good and evil qualities of this temperament, or of its intellectual characteristics.

In the phlegmatic temperament there is a nervous system without much activity, so that the share of talent depends on the active power of the intellect, for it obtains little or no assistance from the nervous energy.

"A deficiency of intellect in the phlegmatic temperament must therefore produce absolute stupidity. The first gradation above stupidity displays a plain, straightforward understanding, entirely destitute of imagination: this forms the class of the *ennuyants*. The next degree shows good sense, with a quicker perception, and a more lively imagination; but still the operations of the intellect are slow, and performed with difficulty, owing to the sluggishness of the brain, and the weakness of the memory. As we advance, the feebleness of the mechanical action is compensated by the increase of intellectual power: its highest degree of perfection shows a clear understanding, a sound judgment, an acute discernment, strong powers of reasoning, and a mind vast and comprehensive, noble and elevated. Here the habit of methodising and analysing assists the memory; the systematic arrangement of the ideas aids the reasoning faculty; the absence of passion gives correctness to the judgment; and the coolness and deliberation with which all the mental operations are performed give clearness to the discernment. Nevertheless the brilliancy of talent displayed in the ardent temperament cannot be attained in the phlegmatic; for, supposing the powers of the intellect to be equal, the latter must always lack the fire and energy which give force and rapidity to the operations of the former."

This sketch is worked out fully and with great ability; we find so great a difficulty of selection, and so impossible to condense or abridge, that we must refer our readers to the book itself.

It may be of interest to our bachelor readers to know that so good an observer warns them if they "value a peaceful life, not to select a short woman, with black hair and a strong fist," but that the phlegmatic temperament, the blue eye, light hair, round limbs, slender shape, and fair complexion, give a man a better chance of a quiet life.

As the ardent temperament is marked by *quickness*, and the phlegmatic by *dulness* in the talents, so they are distinguished by *warmth* and *coldness* in the feelings. And as in the phlegmatic the feelings are most under restraint, the most *faultless* characters belong to it; but still Mrs. Carleton considers the balance of evil belongs to the phlegmatic, as their errors are less excusable, and are derived from a worse origin—selfishness; "and from this foul source proceed the most evil feelings of which our nature is susceptible." The examination of selfishness and the sketch of a purely selfish character, are drawn with a power of mental dissection and demonstration only attained by those who ponder on the evil in their own hearts, and reflect on the excesses to which, if unrestrained, it might lead. The following definition of *sensibility* strikes us as excellent and true:—

"It is, I apprehend, the combination of a quality of the mind, and a peculiarity of the nervous constitution. When a benevolent turn of mind is united to a strong nervous susceptibility, it constitutes genuine sensibility. Benevolence, without delicacy of feeling is mere good nature: susceptibility of feeling, without benevolence, is mere irritability."

Mrs. Carleton's sketch of the four kinds of sensibility in the four different classes of character, is a discriminative piece of practical metaphysical writing of a high order, showing her power of entering into the highest and noblest as well as the weakest feelings of our compound being. But we must conclude with an assurance that the praise we have bestowed is merely from a strict sense of justice to a writer of rare merit, and that our quotations, though enough to justify our opinion, are not the best bits, but the average of the whole composition. We regard it not as a complete disquisition on psychology, but as an important original fragment of that large subject, the result of the attentive observations of very many years of the nervous and mental actions of a highly cultivated mind, and a most delicately organized body: of one whose deeply contemplative nature has been impelled by a ceaseless internal impulse, (the origin of which we cannot trace,) to seek to explain to herself the secret of the connexion of those nerves with that mind; of one who has viewed her thoughts, feelings, emotions, all the valuable experiences of a woman, a wife, and a mother, all her delicate observations of others in the society of the higher classes (for there is internal evidence of this in the delicate handling, the impossibility of a lady's nature to be otherwise than graceful) as experiments to elucidate this her life-problem.

